# Octenyl Succinic Anhydride Starch Alleviates Alcoholic Liver Disease by Modulating Gut Microbiota and Metabolism

**DOI:** 10.3390/nu17172779

**Published:** 2025-08-27

**Authors:** Chang Liu, Tangqian Liu, Rongrong Ma, Xiaohua Pan, Yaoqi Tian

**Affiliations:** 1 State Key Laboratory of Food Science and Resources, Jiangnan University, Wuxi 214122, China; liuchang670250789@jiangnan.edu.cn (C.L.); 1011230114@stu.jiangnan.edu.cn (T.L.); rrma01@jiangnan.edu.cn (R.M.); yqtian@jiangnan.edu.cn (Y.T.); 2 School of Food Science and Technology, Jiangnan University, Wuxi 214122, China; 3 Analysis and Testing Center, Jiangnan University, Wuxi 214122, China

**Keywords:** OSA starch, alcoholic liver disease, gut microbiota, host metabolism

## Abstract

**Background/Objectives**: Alcoholic liver disease (ALD) is intricately linked to gut microbiota dysbiosis and metabolic disturbances along the gut–liver axis. Octenyl succinic anhydride (OSA) starch escapes digestion in the small intestine and ferments in the colon, modulating gut microbiota and metabolism. This study explored the protective effects of OSA starch against ALD and elucidated the underlying gut microbiota–metabolite interactions. **Methods:** A chronic ethanol-fed mouse model was conducted to evaluate the protective effects of OSA starch against ALD, and multi-omics analyses integrating 16S rRNA sequencing, PICRUSt2 functional predictions, and metabolomics were used to reveal potential mechanism. **Results**: OSA starch supplementation in ALD mice significantly reduced liver fat accumulation, lowered the liver index to 4.11%, and restored serum transaminase levels closer to normal. Multi-omics analyses revealed that OSA starch enriched beneficial gut bacteria such as *Faecalibaculum rodentium* and *Bifidobacterium adolescentis*. OSA starch also enhanced microbial metabolic functions, including pyruvate, butanoate, and propanoate metabolism. These shifts were accompanied by regulation of fecal and serum metabolites, including pyruvate, 2-hydroxybutanoic acid, and lactic acid. Structural equation modeling further confirmed that OSA starch ameliorates ALD via coordinated modulation of gut microbiota, microbial functions, metabolites, and serum markers. **Conclusions**: OSA starch protects against alcoholic liver injury by remodeling the gut–liver metabolic network, presenting a promising dietary strategy for ALD.

## 1. Introduction

Alcoholic liver disease (ALD) is a significant contributor to chronic liver injury and mortality worldwide, predominantly driven by prolonged excessive alcohol consumption [1]. The progression of ALD involves hepatic steatosis, underpinned by complex interactions between the liver and the gut [2,3]. Growing evidence highlights the gut–liver axis as a central player in ALD pathogenesis, particularly through gut microbiota dysbiosis and increased intestinal permeability [4]. These alterations facilitate the translocation of various microbial metabolites into the portal circulation, promoting hepatic steatosis and metabolic disruption. Moreover, shifts in gut-derived metabolites—such as short-chain fatty acids, amino acid derivatives, and bile acids—profoundly influence hepatic lipid metabolism, oxidative stress, and immune responses, all of which contribute to ALD progression [5]. ALD is associated with gut bacterial products and their translocation to the liver, although the role of specific microbiota changes remains unclear [6]. Therefore, nutritional interventions targeting the microbiome have emerged as a promising strategy for ALD prevention and treatment [7,8].

Among nutritional interventions, resistant starches have emerged as promising prebiotic agents capable of reshaping gut microbial communities and their metabolic outputs [9,10]. Octenyl succinic anhydride (OSA) starch, as a modified resistant starch, escapes digestion in the small intestine and undergoes fermentation in the colon by commensal bacteria, leading to the production of short-chain fatty acids like butyrate, propionate, and acetate [11,12]. These microbial metabolites play essential roles in enhancing gut barrier integrity and regulating lipid and glucose metabolism. Additionally, resistant starch intake has been linked to favorable shifts in microbial functional pathways, such as those involved in energy metabolism, amino acid synthesis, and bile acid transformation, which collectively support host metabolic health [13,14]. OSA is a commonly used esterification agent that modifies starches, making the derivative amphiphilic and capable of acting as an emulsion stabilizer [15]. Approved as a food additive, OSA-modified starch has undergone rapid global consumption due to its beneficial properties, including reducing fat absorption in the gut by encapsulating fat particles [16]. Given that OSA starch has also been proven to have a highly similar ability to regulate the microbiota and SCFAs, this type of resistant starch may have the potential to prevent ALD. While resistant starch has shown efficacy in improving metabolic diseases like obesity and non-alcoholic fatty liver disease [17,18], OSA starch’s potential to mitigate alcohol-induced liver injury through modulation of gut microbiota and microbial metabolism remains underexplored.

In this study, the protective effects of OSA starch against ALD were systematically evaluated using a chronic alcohol-fed mouse model. Comprehensive assessments, including liver histopathology, serum biochemistry, fecal and serum metabolomics, and gut microbiota profiling via 16S rRNA gene sequencing, were conducted. Functional predictions and pathway analyses were further employed to elucidate microbial metabolic functions influenced by OSA starch. Structural equation modeling was applied to clarify the mechanistic pathways linking OSA starch, gut microbiota, microbial metabolites, and liver health. These findings underscore the potential of OSA starch as a dietary strategy for ALD.

## 2. Materials and Methods

### 2.1. Establishment of Alcoholic Liver Injury Model and Dietary OSA Starch Intervention

A total of 24 male C57BL/6J mice aged eight weeks were purchased from Gem Pharmatech Co., Ltd. (Nanjing, China) and maintained under specific pathogen-free conditions with controlled environment parameters. All animal procedures received ethical approval from the Animal Ethics Committee of Jiangnan University (JN. No 20240930c0641123), following the ARRIVE guidelines for animal research. Mice were randomly assigned to one of three experimental groups (n = 8 per group) using a random number generator to ensure unbiased group allocation: Control, Model, and OSA starch intervention groups. Both the Control and ALD Model groups were fed a standard chow containing native corn starch as the main carbohydrate source. The OSA starch group was provided with a diet where native starch was fully replaced by OSA-modified starch. The dietary formulations followed established protocols from a previous study [19].

To induce alcoholic liver damage, mice in the Model and OSA starch groups received daily oral gavage of ethanol. For the initial 27 days, 42% (*v*/*v*) ethanol was administered, followed by a single dose of 50% (*v*/*v*) ethanol on day 28. Ethanol dosing began at 0.1 mL per 10 g body weight in the first week, and was progressively increased to 0.15 mL/10 g from the third week onward. Control group mice were gavaged with an equivalent volume of sterile water throughout the experiment.

### 2.2. Serum Biochemical Assays

Following an overnight fast, mice were anesthetized via intraperitoneal injection of 2–3% isoflurane. Blood was drawn, and liver tissues were promptly collected for further analysis. Serum was isolated by centrifugation at 3000× *g* for 10 min at 4 °C. Key biochemical markers, including total cholesterol (TC), triglycerides (TG), high-density lipoprotein cholesterol (HDL-C), low-density lipoprotein cholesterol (LDL-C), alanine aminotransferase (ALT), and aspartate aminotransferase (AST), were quantified using a BS-350E automated biochemical analyzer (Myriad Biomedical, Shenzhen, China).

### 2.3. Liver Histology via Hematoxylin and Eosin Staining

Liver specimens were fixed in 4% paraformaldehyde at room temperature for 24 h, dehydrated through a graded ethanol series, cleared with xylene, and embedded in paraffin. Paraffin blocks were sliced into 4 μm sections using a rotary microtome (Leica, IL, USA). The tissue sections were then stained with hematoxylin and eosin (H&E) and examined microscopically for histopathological evaluation.

### 2.4. Untargeted Metabolomics Profiling

Metabolic profiling was conducted using a QTRAP 5500 mass spectrometer (AB SCIEX, Framingham, MA, USA) connected to an ultra-high-performance liquid chromatography (UHPLC) system. Both positive and negative ionization modes of electrospray ionization (ESI) were employed. For extraction, 50 μL/mg of each sample was combined with 400 μL of pre-cooled methanol containing internal standards. Fecal samples were processed with 2–3 stainless steel beads and homogenized using a tissue grinder (60 Hz, 15 s per cycle, 3–4 cycles) under chilled conditions. Samples were then subjected to 15 min of sonication in an ice-water bath and centrifuged at 10,000× *g* for 20 min at 4 °C.

Approximately 300 μL of the supernatant was collected, evaporated to dryness under vacuum at low temperatures, and reconstituted in 100 μL of 20% methanol. The mixture was vortexed, sonicated, and centrifuged again before being transferred to autosampler vials for UHPLC–MS/MS analysis. Chromatographic separation was carried out using a BEH C18 column (1.7 μm, 2.1 × 100 mm, Waters, Milford, MA, USA) with 0.1% formic acid in water as mobile phase A and acetonitrile as mobile phase B for positive mode. For negative ion mode, an HSS T3 column (1.8 μm, 2.1 × 100 mm, Waters, Milford, MA, USA) was utilized. The column temperature was maintained at 50 °C. Data acquisition and initial analysis were performed using Analyst software 1.7.2 (AB SCIEX, Framingham, MA, USA). The identity of the metabolites was confirmed based on the MS/MS spectra, comparing the observed fragmentation patterns with available reference spectra in the database. This included matching retention times and the specific mass-to-charge values, as well as interpreting the fragmentation pathways for structural confirmation. Additionally, the metabolite analysis was further validated by comparing the results with data available on MetaboAnalyst 5.0, which provides comprehensive information on known metabolites and their fragmentation profiles [20].

### 2.5. 16S rRNA Gene Sequencing and Microbial Functional Prediction

Genomic DNA from fecal samples was extracted with the TIANamp Stool DNA Kit (DP328, TIANGEN, Beijing, China), following the manufacturer’s instructions. The quality and quantity of DNA were assessed via a NanoDrop spectrophotometer (Thermo Fisher Scientific, Waltham, MA, USA) and agarose gel electrophoresis. The V3–V4 regions of the bacterial 16S rRNA gene were amplified using specific primers with Illumina adapters. Purified PCR products were quantified and pooled in equimolar amounts. Libraries were prepared using the TruSeq DNA PCR-Free Library Prep Kit (Illumina, San Diego CA, USA), and sequencing was conducted on the Illumina NovaSeq 6000 platform (paired-end, 2 × 250 bp).

Raw sequences underwent quality filtering with Trimmomatic to remove adapters and low-quality reads, and merged using FLASH. Operational taxonomic units (OTUs) were clustered at 97% sequence similarity via USEARCH. Taxonomic classification was performed against the SILVA database (version 138). Microbial functional potential was predicted using PICRUSt2 to infer KEGG Orthologs (KOs) based on 16S rRNA profiles. All sequencing and computational analyses were completed by Wekemo Tech Group Co., Ltd. (Shenzhen, China).

### 2.6. Statistical and Computational Analyses

Data are expressed as mean ± standard deviation (SD). Statistical analyses were carried out using GraphPad Prism 7.0 (GraphPad Software Inc., San Diego, CA, USA). Differences between groups were evaluated by one-way analysis of variance (ANOVA) with Dunnett’s post hoc test. A *p*-value < 0.05 was considered statistically significant (* *p* < 0.05). Differential microbial taxa were identified using linear discriminant analysis effect size (LEfSe) on the Galaxy platform (https://usegalaxy.org/).

## 3. Results and Discussion

### 3.1. OSA Starch Ameliorates Hepatic Injury and Lipid Dysregulation in ALD Mice

**Figure 1:**
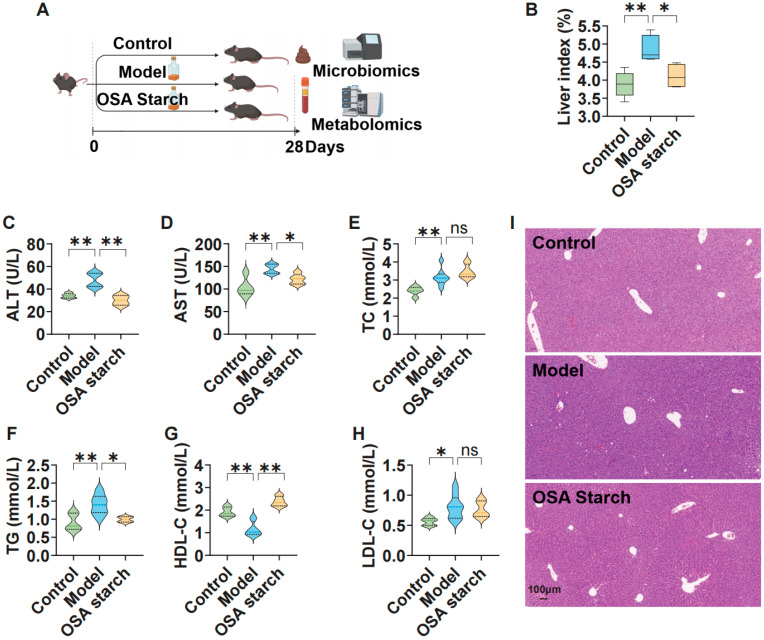
OSA starch ameliorates hepatic injury and lipid dysregulation in AL mice. (**A**) Schematic representation of the experimental protocol. (**B**) Liver index. (**C**,**D**) Serum ALT and AST levels. (**E**,**F**) Serum TC and TG levels. (**G**,**H**) Serum HDL-C and LDL-C levels. (**I**) Representative liver histology with H&E staining. Data are presented as mean ± SD (n = 8). Statistical significance was determined by one-way ANOVA with Dunnett’s post hoc test: ns, not significant *p* > 0.05, * *p* < 0.05, ** *p* < 0.01.

To evaluate the protective effects of OSA starch on ALD, a chronic alcohol feeding model in mice, together with a 28-day intervention with OSA starch, was established (Figure 1A). Alcohol exposure significantly elevated the liver index, indicating hepatomegaly as a consequence of fat accumulation (*p* < 0.01, Figure 1B) [21]. OSA starch supplementation markedly reduced the liver index compared to the ALD Model group (*p* < 0.05), suggesting mitigation of alcohol-induced liver enlargement. This reduction corresponds with a significant decrease in serum alanine aminotransferase (ALT) and aspartate aminotransferase (AST) levels (*p* < 0.01 and *p* < 0.05, respectively; Figure 1C,D), key biomarkers for liver injury [22]. Consistently, histological examination revealed pronounced steatosis and hepatocellular ballooning in the ALD Model group, whereas OSA starch intervention substantially alleviated these pathological features, maintaining liver architecture closer to that of controls (Figure 1I). These outcomes align with prior studies indicating that dietary fibers can modulate the gut–liver axis, thereby alleviating alcohol-induced hepatic injury [23].

In parallel, alcohol exposure disrupted lipid metabolism, as reflected by elevated total cholesterol (TC), triglycerides (TG), and low-density lipoprotein cholesterol (LDL-C), alongside decreased high-density lipoprotein cholesterol (HDL-C) (Figure 1E–H) [24,25]. OSA starch supplementation significantly lowered TG levels (*p* < 0.05) and restored HDL-C levels (*p* < 0.01), both of which are critical in mitigating the progression of steatosis and cardiovascular risk. Interestingly, OSA starch did not significantly alter TC or LDL-C levels compared to the ALD Model group. Together, these findings demonstrate that OSA starch exerts benefits on liver morphology, function, and lipid metabolism in ALD.

### 3.2. OSA Starch Intervention Reshapes Gut Microbiota Composition in ALD Mice

**Figure 2:**
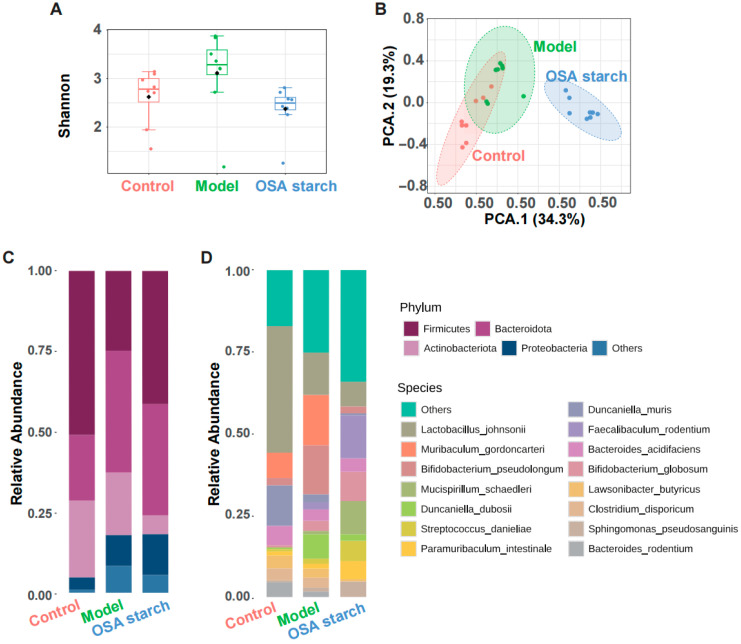
OSA starch intervention reshapes gut microbiota composition in ALD mice. (**A**) Alpha diversity indices—Shannon. (**B**) PCA based on Bray–Curtis dissimilarity reveals distinct clustering. (**C**) Gut microbiota composition at the phylum level. (**D**) Gut microbiota composition at the species level.

To investigate the regulatory effects of OSA starch on gut microbial dysbiosis induced by alcohol exposure, alpha diversity and microbial community structure in ALD mice were assessed [26]. Shannon indices (Figure 2A) revealed that chronic alcohol consumption increased species richness, reflecting an unstable gut microbial species [27]. Interestingly, OSA starch supplementation attenuated the ethanol-induced increase in microbial species richness, indicating a selective regulation of the microbial composition. Principal component analysis (PCA) based on Bray–Curtis distance (Figure 2B) demonstrated a clear segregation of microbial profiles, with the OSA starch group distinctly clustering away from the ALD model, indicating a pronounced shift in microbial structure attributable to the dietary intervention.

At the taxonomic level, phylum-level analysis (Figure 2C) showed that alcohol exposure disrupted the balance between Firmicutes and Bacteroidota. OSA starch partially restored this ratio, primarily by reducing the relative abundance of Proteobacteria, a phylum often associated with barrier dysfunction [28]. At the phylum level, alcohol exposure increased the relative abundance of Proteobacteria while decreasing Firmicutes, consistent with dysbiosis patterns commonly observed in liver injury (Figure 2C) [29]. Species-level profiling (Figure 2D) revealed that OSA starch increased beneficial microbes such as *Faecalibaculum rodentium* and *Bifidobacterium adolescentis*, while reducing the abundance of potentially pathogenic taxa like *Muribaculum gordoncarteri*. These compositional changes are consistent with the known prebiotic effects of resistant starches, which modulate the gut microbiota to enhance host metabolic and immune health [9,30]. Collectively, these results suggest that OSA starch ameliorates alcohol-induced gut dysbiosis through microbiome modulation.

### 3.3. OSA Starch Modulates Key Gut Microbial Taxa and Their Functional Profiles in ALD Mice

**Figure 3:**
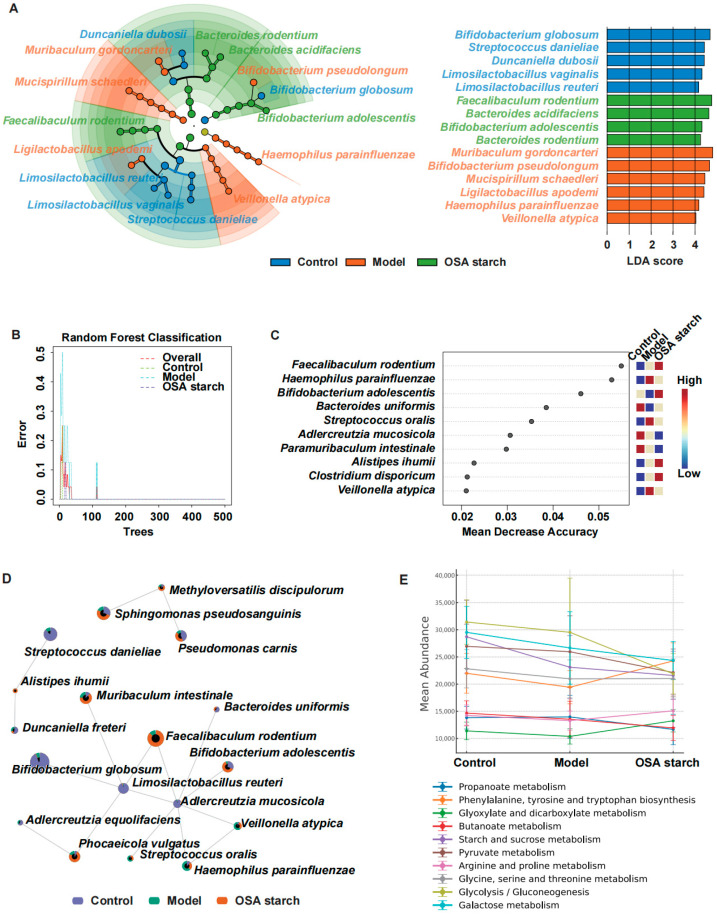
OSA starch modulates key gut microbial taxa and their functional profiles in ALD mice. (**A**) LEfSe cladogram and LDA score of differential species. (**B**) Random forest classification error rates for overall and individual groups. (**C**) Top microbial features ranked by mean decrease accuracy contributing to group discrimination. (**D**) Co-occurrence network of differential gut microbes colored by group prevalence. (**E**) Predicted KEGG metabolic pathways (Level 3) of gut microbiota across groups.

Comprehensive analysis of gut microbiota alterations induced by OSA starch intervention in ALD mice revealed significant shifts in bacterial composition and diversity. LEfSe analysis identified distinct microbial biomarkers across experimental groups (Figure 3A). The ALD Model group exhibited an enrichment of potentially pathogenic species such as *Haemophilus parainfluenzae*, *Veillonella atypica*, and *Muribaculum gordoncarteri*, which have been implicated in gut barrier dysfunction and inflammation [31,32]. Conversely, OSA starch supplementation selectively enriched beneficial taxa, including *Faecalibaculum rodentium*, *Bacteroides acidifaciens*, and *Bifidobacterium adolescentis*. These species are recognized as producers of short-chain fatty acids, particularly butyrate, known to reinforce intestinal integrity and exert anti-inflammatory effects, potentially contributing to the alleviation of ALD pathology [23,33,34].

Random forest classification further validated the discriminative microbial features between groups, achieving low classification error rates (Figure 3B). The most influential taxa contributing to group separation, as indicated by the mean decrease in accuracy, included *Faecalibaculum rodentium*, *Haemophilus parainfluenzae*, and *Bifidobacterium adolescentis* (Figure 3C). These key species are involved in metabolic pathways essential for maintaining host lipid homeostasis and modulating immune responses [35,36]. Co-occurrence network analysis (Figure 3D) demonstrated that *Bifidobacterium adolescentis* and *Faecalibaculum rodentium* formed central nodes within the microbial ecosystem of OSA starch-treated mice, suggesting that OSA starch fosters a more interconnected microbial community structure.

Functional predictions based on PICRUSt2 and KEGG pathway analysis revealed that OSA starch intervention notably influenced microbial metabolic potential (Figure 3E). Specifically, pathways associated with propanoate metabolism, glycolysis/gluconeogenesis, and galactose metabolism were reduced in the OSA starch group, which linked to metabolic disturbances in ALD [37]. In contrast, pathways such as glyoxylate and dicarboxylate metabolism were relatively enhanced, indicating a metabolic shift towards improved energy efficiency and substrate utilization. These microbial functional adaptations align with the biochemical improvements observed in liver function and lipid profiles, supporting the role of OSA starch in mitigating ALD progression through targeted modulation of the gut microbiome and its metabolism.

### 3.4. Key Gut Microbial Metabolic Pathways Modulated by OSA Starch in ALD Mice

Functional pathway analysis based on PICRUSt2 prediction revealed that OSA starch notably regulated gut microbial metabolic functions centered on carbon metabolism, including the pyruvate, glyoxylate, and dicarboxylate, butanoate, and propanoate metabolism pathways (Supplementary Figure S1). In the pyruvate metabolism pathway, several critical enzymes responsible for converting pyruvate into acetyl-CoA, lactate, and other intermediates were significantly altered (Supplementary Figure S1A). Hepatic pyruvate metabolism is critical and complementary for maintenance of antioxidant capacity [38]. Additionally, modulation of glyoxylate and dicarboxylate metabolism suggests enhanced microbial capacity for the glyoxylate cycle (Supplementary Figure S1B), which is pivotal in maintaining energy efficiency under metabolic stress conditions commonly associated with liver diseases [39].

Moreover, the butanoate and propanoate metabolism pathways were distinctly influenced, as evidenced by the differential regulation of enzymes involved in the synthesis of key short-chain fatty acids, such as butyrate and propionate (Supplementary Figure S1C,D). Short-chain fatty acids are critical mediators of gut–liver axis health, with butyrate known to strengthen intestinal barrier integrity and propionate contributing to lipid metabolism regulation [40,41]. The attenuation of propanoate metabolism alongside the remodeling of butanoate pathways may help restore short-chain fatty acid homeostasis disrupted in ALD. This metabolic reconfiguration in the gut microbiota highlights a potential mechanism by which OSA starch exerts its protective effects against hepatic injury via microbial metabolic modulation.

### 3.5. OSA Starch Reshapes Fecal Metabolome and Modulates Key Metabolic Pathways in ALD Mice

**Figure 4:**
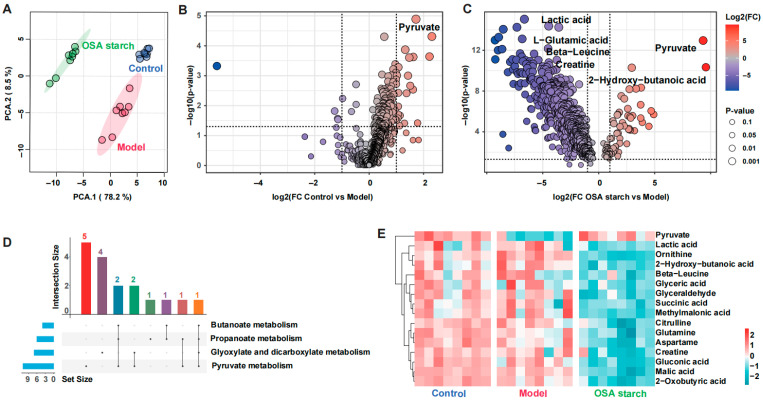
OSA starch reshapes the fecal metabolome in ALD mice. (**A**) PCA score plot of fecal metabolome showing group separation. (**B**) Volcano plot of differential metabolites between Control and Model groups. (**C**) Volcano plot of differential metabolites between OSA starch and Model groups. (**D**) Upset plot indicating metabolite overlap among four key pathways (pyruvate, propanoate, butanoate, glyoxylate/dicarboxylate metabolism). (**E**) Heatmap of representative differential metabolites across groups.

Untargeted fecal metabolomics revealed a distinct metabolic reprogramming following OSA starch intervention in ALD mice. PCA demonstrated clear separation between the Control, Model, and OSA starch groups, reflecting the overall impact of OSA starch on metabolic profiles (Figure 4A). The volcano plots identified pyruvate accumulation as a hallmark of ALD progression, with the Model group showing significant decreases compared to Control (Figure 4B). Conversely, OSA starch supplementation not only sustained elevated pyruvate and 2-hydroxy-butanoic acid levels but also substantially reduced the abundance of lactic acid, L-glutamic acid, beta-leucine, and creatine—metabolites linked to disrupted amino acid and energy metabolism (Figure 4C). These metabolic shifts suggest that OSA starch may redirect pyruvate flux away from lactate production and abnormal amino acid turnover, promoting more efficient energy homeostasis. Further analysis using Upset plots identified metabolites converging on four pivotal metabolic pathways: pyruvate metabolism, propanoate metabolism, butanoate metabolism, and glyoxylate and dicarboxylate metabolism (Figure 4D). These pathways are central to maintaining carbon flow between glycolysis, the TCA cycle, and short-chain fatty acid production [42]. The heatmap clustering emphasized the normalized metabolic landscape in the OSA starch group relative to the Model (Figure 4E).

**Figure 5:**
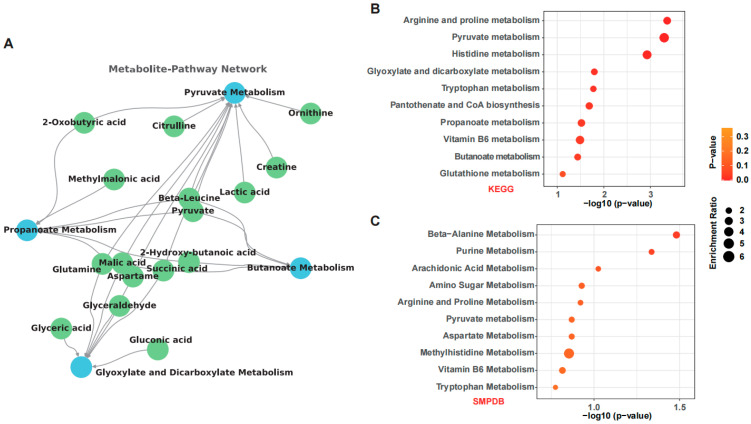
OSA starch modulates key metabolic pathways in ALD mice. (**A**) Metabolite–Pathway network connecting key metabolites to their respective metabolic pathways. Blue represents metabolic pathways, and green represents metabolic metabolites. (**B**) KEGG pathway enrichment analysis of altered metabolites (*p* < 0.01). (**C**) SMPDB pathway enrichment analysis of altered metabolites (*p* < 0.01). Red represents altered metabolic pathways in KEGG Database and SMPDB database, respectively.

A detailed metabolite–pathway interaction network (Figure 5A) mapped the relationships between these four key pathways and their representative metabolites. Pyruvate metabolism emerged as a central hub, directly connected to citrulline, ornithine, creatine, lactic acid, and beta-leucine, among others. This centrality underscores pyruvate’s critical role as a metabolic crossroad linking amino acid biosynthesis and energy production [43]. Additionally, propanoate, butanoate, and glyoxylate metabolism were interconnected through shared metabolites such as glutamine, glyceric acid, and malic acid, highlighting the intricate metabolic crosstalk restored by OSA starch intervention. KEGG enrichment analysis further revealed significant regulation of pathways involved in arginine and proline metabolism, pyruvate metabolism, histidine metabolism, and glyoxylate pathways (Figure 5B), which are also relevant to hepatic and intestinal health [44]. Complementarily, SMPDB enrichment confirmed involvement of beta-alanine, purine, and arachidonic acid metabolism (Figure 5C), suggesting broader impacts on amino acid turnover and inflammatory responses. Collectively, these findings suggest that OSA starch exerts hepatoprotective effects by orchestrating the gut-derived metabolic networks.

### 3.6. OSA Starch Improves Serum Bile Acid Profiles in ALD Mice

**Figure 6:**
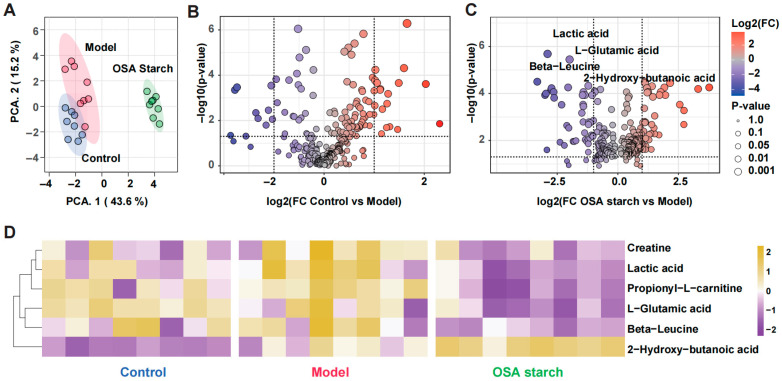
OSA starch improves serum metabolism profiles in ALD mice. (**A**) PCA based on untargeted serum metabolomics. (**B**) Volcano plot of differentially expressed serum metabolites between the Control and Model groups. (**C**) Volcano plot of differentially expressed serum metabolites between OSA starch and Model groups. (**D**) Heatmap showing the relative abundance of significantly altered metabolites.

Untargeted serum metabolomics revealed that OSA starch supplementation significantly remodeled the serum metabolic landscape in ALD mice. PCA illustrated distinct clustering of the Control, Model, and OSA starch groups, indicating that OSA starch markedly shifted the serum metabolome towards a healthier profile (Figure 6A). Differential analysis between the OSA starch and Model groups identified several metabolites that were significantly altered, particularly those associated with amino acid metabolism and energy balance (Figure 6B). Key metabolites such as lactic acid, L-glutamic acid, beta-leucine, creatine, and propionyl-L-carnitine showed substantial reductions following OSA starch intervention, suggesting improved mitochondrial function and reduced metabolic stress [45].

Heatmap visualization further highlighted that OSA starch restored the serum levels of these representative metabolites (Figure 6D). The reduction in lactic acid and glutamic acid implies mitigation of anaerobic glycolysis and excitotoxicity, respectively, both of which are aggravated in liver disease [46,47]. Additionally, the decrease in beta-leucine and propionyl-L-carnitine may reflect a normalization of branched-chain amino acid metabolism and fatty acid oxidation, respectively, further indicating metabolic reprogramming favoring hepatic health [48]. These findings are consistent with previous reports where targeting amino acid and lipid metabolism alleviated liver injury and improved metabolic resilience [18]. Overall, the serum metabolomic data support the hypothesis that OSA starch exerts protective effects against ALD by rebalancing energy production.

### 3.7. OSA Starch Modulates Gut Microbiota–Metabolite–Host Axis to Alleviate ALD

**Figure 7:**
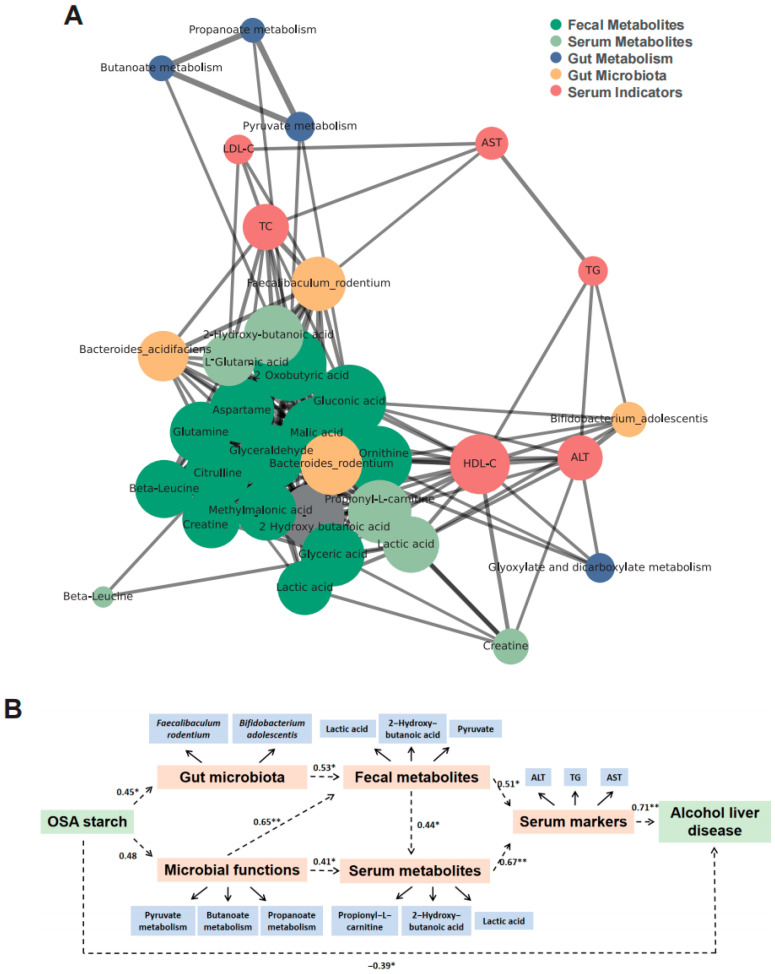
OSA starch modulates gut microbiota–metabolite–host axis to alleviate ALD. (**A**) Interaction network depicting relationships among gut microbiota, microbial metabolism, fecal metabolites, serum metabolites, and serum indicators. (**B**) SEM illustrating the pathways from OSA starch intervention through gut microbiota, microbial functions, fecal and serum metabolites, and serum markers to alcoholic liver disease outcome. * *p* < 0.05, ** *p* < 0.01.

To further elucidate the mechanism underlying the beneficial effects of OSA starch on ALD, a comprehensive multi-layer network was constructed integrating gut microbiota, microbial functions, fecal and serum metabolites, and clinical serum indicators. The interaction network (Figure 7A) revealed *Faecalibaculum rodentium* and *Bifidobacterium adolescentis* as central bacterial nodes connected to key metabolic pathways, including butanoate and propanoate metabolism, and to signature fecal metabolites such as pyruvate, lactic acid, and 2-hydroxy-butanoic acid. These metabolites exhibited strong links with serum markers like ALT, AST, and TG, suggesting that gut microbial modulation impacts host metabolic health. Additionally, microbial functional pathways including pyruvate and propanoate metabolism were identified as pivotal nodes linking gut microbiota alterations to metabolic outputs, reinforcing the microbiota–metabolite–host axis hypothesis.

A structural equation model (SEM) was constructed using AMOS software version 28 (Armonk, NY, USA) to quantify the direct and indirect pathways involved (Figure 7B). The final model exhibited good fit to the data, with fit indices of RMSEA = 0.065 and CFI = 0.975, indicating an acceptable model fit. OSA starch positively regulated gut microbiota (path coefficient = 0.45, *p* < 0.01) and microbial functions (0.48, *p* < 0.01), which sequentially affected fecal metabolites (0.53, *p* < 0.01) and serum metabolites (0.41, *p* < 0.05). Both fecal and serum metabolites significantly influenced serum markers (0.51, *p* < 0.01 and 0.44, *p* < 0.05, respectively). OSA starch exhibited a strong negative correlation with ALD progression (−0.39, *p* < 0.05). These findings suggest that the gut microbiota–metabolite axis serves as a critical mediator of OSA starch’s therapeutic benefits in ALD.

Network and SEM analyses strongly support the hypothesis that OSA starch alleviates ALD by modulating the gut microbiota–metabolite–host axis. This central role of the gut microbiota aligns with the protective mechanisms attributed to other prebiotics and dietary fibers in ALD research, such as polysaccharides [49]. The unique structure and physical and chemical properties of OSA starch may affect the way this regulatory mechanism is realized, thereby influencing the succession of microorganisms and the availability of ecological niches. This indicates that although the core protective axis (gut–liver communication) is the common goal, different prebiotic fibers participate in it through subtle mechanisms.

While these findings show the beneficial effects of OSA starch on the gut–liver axis in ALD mice, several limitations exist. First, murine models differ from humans in alcohol metabolism and disease progression, limiting translation to human ALD. Second, the 28-day intervention may not reflect long-term OSA starch effects or chronic ALD progression, warranting longer studies. Third, replacing native starch entirely with OSA starch does not mimic typical human diets, suggesting that future studies should examine OSA starch in more complex dietary contexts.

## 4. Conclusions

In conclusion, this study suggests that dietary OSA starch may ameliorate alcoholic liver disease by orchestrating a gut microbiota–metabolite–host axis. OSA starch selectively enriched beneficial gut microbes such as *Faecalibaculum rodentium* and *Bifidobacterium adolescentis*, which appeared associated with enhanced microbial metabolic functions, including pyruvate, butanoate, and propanoate metabolism. Consistent with this, favorable changes in fecal and serum metabolites (e.g., elevated pyruvate and reduced lactic acid), along with improvements in serum lipid profiles and hepatic injury markers, were observed. Structural equation modeling indicates that OSA starch exerts its protective effects through a multi-level regulatory cascade spanning the gut microbiota, microbial functions, metabolic intermediates, and serum indicators. These findings highlight the potential of OSA starch as a dietary intervention for alcoholic fatty liver disease by modulating the gut–liver metabolic axis.

## Data Availability

The Dataset available on request from the authors.

## Supplementary Materials

The following supporting information can be downloaded at: https://www.mdpi.com/article/10.3390/nu17172779/s1, Figure S1: Key Gut Microbial Metabolic Pathways Modulated by OSA Starch in ALD mice.

## Abbreviations

The following abbreviations are used in this manuscript:

ALD	Alcoholic liver disease
OSA	Octenyl succinic anhydride
