# Supplementary material for: Octenyl Succinic Anhydride Starch Alleviates Alcoholic Liver Disease by Modulating Gut Microbiota and Metabolism
Source: Nutrients. 2025 Aug 27;17(17):2779. doi: 10.3390/nu17172779 (PMC12430434; doi:10.3390/nu17172779)
Supplement: Supplementary file 1 [file nutrients-3791375-supplementary.pdf › nutrients-3791375-supplementary.pdf]

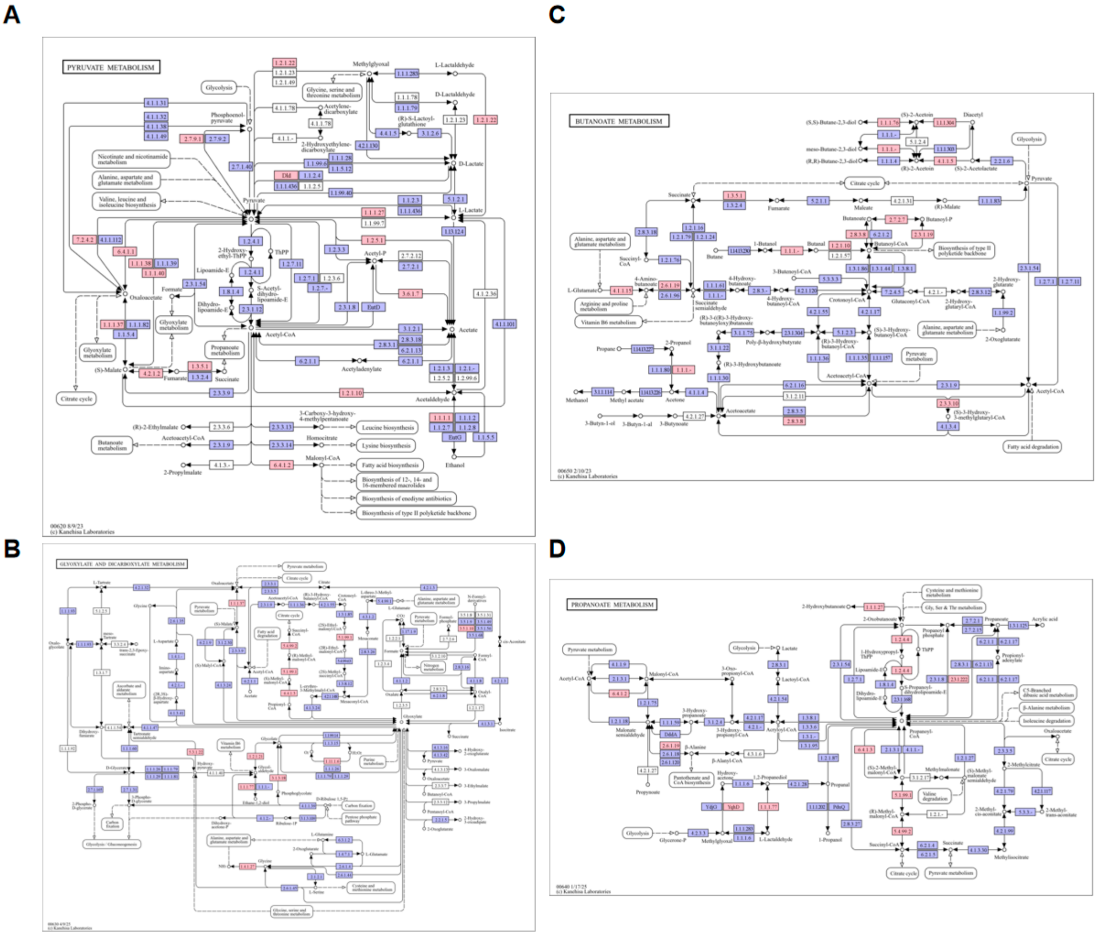

Supplement Figure S1 Key Gut Microbial Metabolic Pathways Modulated by OSA Starch in ALD mice

(A) Pyruvate Metabolism (ko00620)

(B) Glyoxylate and Dicarboxylate Metabolism (ko00630)

(C) Butanoate Metabolism (ko00650)

(D) Propanoate Metabolism (ko00640)

Significantly regulated enzymes (p < 0.01) are highlighted in pink.
